# Enhancing the magnetic anisotropy of maghemite nanoparticles via the surface coordination of molecular complexes

**DOI:** 10.1038/ncomms10139

**Published:** 2015-12-04

**Authors:** Yoann Prado, Niéli Daffé, Aude Michel, Thomas Georgelin, Nader Yaacoub, Jean-Marc Grenèche, Fadi Choueikani, Edwige Otero, Philippe Ohresser, Marie-Anne Arrio, Christophe Cartier-dit-Moulin, Philippe Sainctavit, Benoit Fleury, Vincent Dupuis, Laurent Lisnard, Jérôme Fresnais

**Affiliations:** 1 Sorbonne Universités, UPMC Univ Paris 06, UMR 8234, PHENIX, CNRS, F-75005 Paris, France; 2 Institut de Minéralogie, de Physique des Matériaux et de Cosmochimie, UMR 7590, CNRS, UPMC, IRD, MNHN, F-75005 Paris, France; 3 Synchrotron SOLEIL, L'Orme des Merisiers, Saint-Aubin—BP 48, 91192 Gif-sur-Yvette, France; 4 Sorbonne Universités, UPMC Univ Paris 06, UMR 7197, LRS, F-94200 Ivry-sur-Seine, France; 5 CNRS, UMR 7197, Laboratoire de Réactivité de Surface, F-94200 Ivry-sur-Seine, France; 6 Institut des Molécules et Matériaux du Mans CNRS UMR-6283, Université du Maine, F-72085 Le Mans, France; 7 Sorbonne Universités, UPMC Univ Paris 06, UMR 8232, IPCM, F-75005 Paris, France; 8 CNRS, UMR 8232, Institut Parisien de Chimie Moléculaire, F-75005 Paris, France

## Abstract

Superparamagnetic nanoparticles are promising objects for data storage or medical applications. In the smallest—and more attractive—systems, the properties are governed by the magnetic anisotropy. Here we report a molecule-based synthetic strategy to enhance this anisotropy in sub-10-nm nanoparticles. It consists of the fabrication of composite materials where anisotropic molecular complexes are coordinated to the surface of the nanoparticles. Reacting 5 nm γ-Fe_2_O_3_ nanoparticles with the [Co^II^(TPMA)Cl_2_] complex (TPMA: tris(2-pyridylmethyl)amine) leads to the desired composite materials and the characterization of the functionalized nanoparticles evidences the successful coordination—without nanoparticle aggregation and without complex dissociation—of the molecular complexes to the nanoparticles surface. Magnetic measurements indicate the significant enhancement of the anisotropy in the final objects. Indeed, the functionalized nanoparticles show a threefold increase of the blocking temperature and a coercive field increased by one order of magnitude.

## Abstract (web summary)

The enhancement of the magnetic anisotropy of superparamagnetic nanoparticles may increase their functionality. Here, the authors report that the functionalization of maghemite nanoparticles with cobalt(II) complexes enhances their magnetic anisotropy, and subsequently blocking temperature and coercive field.

## Graphical abstract

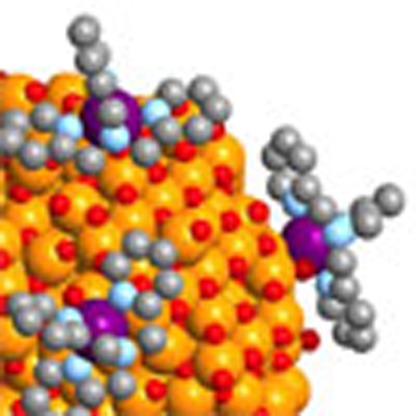

In single-domain superparamagnetic nanoparticles, magnetic anisotropy has a direct impact on the magnetization, its remanence, reversal and relaxation. Magnetic anisotropy is therefore a key parameter in the preparation of magnetic nanocrystals designed for high-density data storage applications or for medical applications^1^,^2^,^3^,^4^,^5^,^6^,^7^. For such applications, achieving a controlled modulation of the magnetic anisotropy for a given size of crystals represents thus one of the most efficient ways to improve and tune their magnetic properties. For example, the optimization of the specific loss power of nanocrystals for magnetic hyperthermia and the comprehension of the interplay between magnetic anisotropy and magnetization is of crucial importance for applications in nanomedicine^8^,^9^. On the other hand, the fabrication of small magnetic nanocrystals displaying high blocking temperature while maintaining magnetic bistability—that is, large coercive fields—remains a considerable challenge aimed at overtaking the so-called superparamagnetic limit and increasing data storage densities^10^.

Different chemical approaches have been used so far to alter the magnetic anisotropy of single-domain magnetic nanoparticles: particle doping or formation of alloys^11^,^12^,^13^,^14^, coordination of solvent or organic molecules to the nanoparticles surface^15^,^16^,^17^, preparation of core-shell systems incorporating highly anisotropic components^8^,^9^,^12^,^18^,^19^,^20^,^21^,^22^,^23^ and insertion of the nanoparticles into magnetic host matrices^10^,^24^.

Herein, we report a strategy for the preparation of anisotropically enhanced magnetic nanoparticles. Our synthetic strategy is based on the direct coordination of magnetic molecular complexes to the surface of the nanoparticles. Using molecular complexes as the enhancing unit is a straightforward approach to improve the nanoparticles properties and it also represents a very advantageous functionalization tool. Indeed, coordination chemistry with its great versatility allows the design and the use of specific complexes where both the local ion anisotropy and the nature of the coordination sphere are controlled. The use of coordination complexes to achieve surface functionalization will also prevent diffusion of the ions into the particles. Decorating magnetic nanoparticles with molecular complexes is thus equivalent to building a new surface with ions whose environment and hence local anisotropy is predetermined. This represents an efficient way to not only modify but also tune the nanoparticles surface anisotropy, which is known to contribute greatly to the whole magnetic anisotropy and actually be one of its most influential components in small systems^25^,^26^. Furthermore, investigations motivated by the study of the possible interactions between two magnetic components in hybrid materials made from inorganic nanomaterials and coordination compounds remain scarce^27^. In another type of multi-scale hybrid system, it has been shown that the magnetization relaxation of deposited mononuclear complexes can be influenced by a magnetic substrate^28^. Our work, however, is motivated by the opposite phenomena, that is the influence of the complexes on the magnetic behaviour of the nanoparticles. We demonstrate here that the grafting of an adequate magnetic complex—even for a low quantity—on a superparamagnetic nanoparticle leads to a massive improvement of the magnetic properties.

## Results

### Strategy

In combining small superparamagnetic nanoparticles and magnetic molecular complexes we wished to efficiently transmit the anisotropy of the complex to the particles and thus achieve the modulation of the particles magnetic anisotropy. As the magnetic moment originating from mononuclear complexes (few Bohr magnetons) is weak compared to that of a particle (thousands of Bohr magnetons), weak interactions or electrostatic interactions between the complexes and the particles were not suitable for the occurrence of a significant magnetic effect. Therefore, we have targeted the formation of a chemical bond between the complexes and the particles as the best way to promote a strong exchange pathway, namely, a coordination bridge between the metal ions of the complexes and the ones located at the surface of the particles. To successfully coordinate complexes at the surface of nanoparticles and yield an anisotropically enhanced system, we have followed three prerequisites.

**Figure 1:**
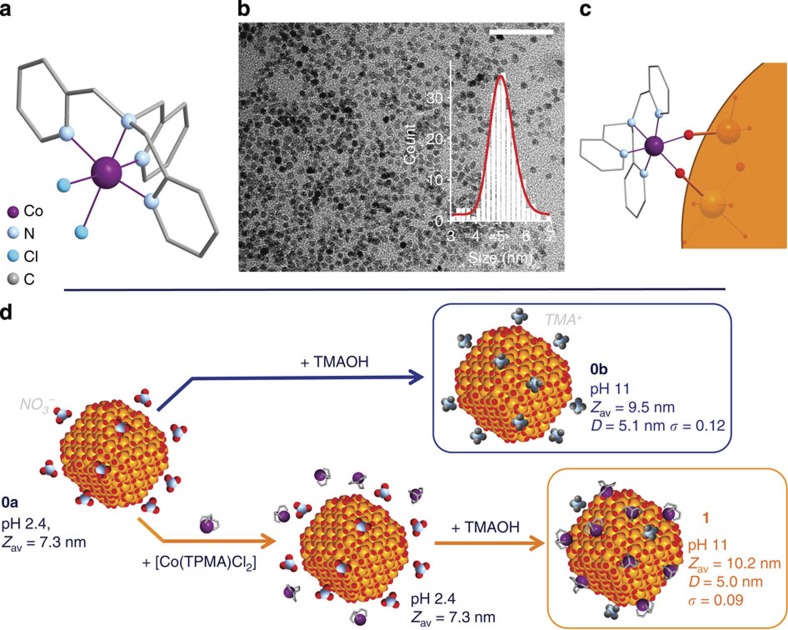
Enhancing molecular complex and functionalized maghemite nanoparticles. (**a**) Representation of the [Co(TPMA)Cl_2_] complex used to enhance the magnetic anisotropy of the γ-Fe_2_O_3_ nanoparticles. (**b**) TEM image of the γ-Fe_2_O_3_ nanoparticles functionalized with the cobalt(II) complex: **1** (5.0 nm, *σ*=0.09) and (**c**) schematic view of the coordination of the complex with the iron ions. (**d**) Synthesis scheme with measured pH values, hydrodynamic diameters (*Z*_av_), sizes (*D*) and distributions (*σ*).

First, we have selected a magnetic complex made with a polydentate ligand and bearing labile groups. Such a ligand should warrant the stability and the geometry of the complex while the labile groups will promote the coordination of the complex to the particle surface. In this work, we have chosen the complex [Co(TPMA)Cl_2_] (ref. 29; TPMA, tris(2-pyridylmethyl)amine, Fig. 1a). With its intrinsic magnetocrystalline anisotropy, cobalt(II) was an obvious choice of metal ion^30^,^31^,^32^. The tetradentate TPMA ligand occupies four positions in the cobalt(II) coordination sphere leaving two *cis*-coordinated chloride groups that are easily substitutable. As the geometry of the mononuclear cobalt(II) complex allows no more than one layer of complex at the surface of the particles, this approach guarantees a low increase of the nanoparticle size.

Second, we have chosen magnetic nanoparticles possessing coordinating atoms at their surface that could be synthesized without any stabilizing organic species. The use of bare nanoparticles is indeed necessary to promote the approach of the complexes to the particles surface and then the coordination reaction. The well-known Massart procedure along with a thorough size sorting procedure allows the synthesis of maghemite nanoparticles γ-Fe_2_O_3_ with a relatively low polydispersity and a small size^33^,^34^. The colloidal solutions of the particles are stabilized by the pH-dependent surface charge (positive or negative under acidic or basic conditions, respectively) that prevents the use of any stabilizing organic species.

Finally, it was crucial to maintain colloidal stability during and after the coordination reaction of the complexes at the nanoparticles surface. We took special care to ensure that no aggregation of the particles was occurring in the solution, since it would have led to undesirable magnetic dipolar interactions. Indeed, such inter-particle interactions would have masked the actual impact of the complexes on the nanoparticles magnetic behaviour.

### Synthesis and characterization

We have used small maghemite nanoparticles in acidic colloidal solution (sample **0a**, *D*_0_=5.1 nm, *σ*=0.12; refs 33, 34). In a first step, the [Co(TPMA)Cl_2_] complex is added at room temperature to the nanoparticles acidic solution. The number of added complexes has been varied from ∼3 to 210 per nanoparticle. At this stage, no iono-covalent bond between the complexes and the particles surface is expected, albeit supramolecular interactions cannot be ruled out. There are furthermore no sign of increase of the hydrodynamic diameter in dynamic light scattering (DLS). A single peak is detected and it remains close to the value observed for the bare acidic particles **0a** (*Z*_av_=7.3 nm). In the second step, the condensation of the complexes at the surface of the particles takes place by a brutal modification of the pH: from 2.4 to 11 with the addition of a concentrated solution of tetramethylammonium hydroxide (TMAOH), to give the functionalized nanoparticles (Fig. 1c). The very quick crossing of the zero point charge (pH 7–8) allows the conservation of the colloidal stability. An increase of the hydrodynamic diameter is observed after the condensation reaction, up to *Z*_*av*_=10.2 nm when ∼85 complexes were added per nanoparticle in the first step (Supplementary Fig. 1 and Supplementary Table 1). Sample **1**, which corresponds to the nanoparticles functionalized by the addition of ca. 60 complexes per particle in the first synthesis step, shows a similar increase. This can be ascribed to the coordination of the complexes and to the presence around the particles of TMA^+^ counterions, which accompany the modification of the nature of the surface charge (from positively to negatively charged). Indeed, for bare nanoparticles, DLS shows a similar increase of the hydrodynamic diameter (from 7.3 to 9.5 nm) when performing the brutal pH change in the absence of complexes: passing from **0a** (pH 2.4) to the basified colloidal solution (sample **0b**, pH 11) with the addition of TMAOH. For the functionalized nanoparticles, the absence of any additional peaks in DLS indicates that there are neither aggregation of particles nor side nucleation of cobalt oxide—that could have occurred were the complexes unstable. No evolution of the single peak has been observed over weeks. The addition of >85 complexes per particle induces a dramatic increase of the hydrodynamic diameter, followed by the flocculation of the particles. The latter is probably caused by the loss of the electrostatic repulsion-induced stabilization that should accompany the increase of the grafting rate. In the following we will focus on **0b** and **1**.

Transmission electron microscopy (TEM) indicates that very similar sizes and distributions are observed for **0b** and **1** (5.1 nm, *σ*=0.12 and 5.0 nm, *σ*=0.09, respectively; Fig. 1b and Supplementary Fig. 2). Along with the DLS experiments, this supports the absence of aggregation or of higher size particles. It also indicates that the functionalization has a negligible effect on the size of the objects. X-ray powder pattern analysis shows that **0b** and **1** both display the cubic structure of the maghemite (*Fd-3m*) while the estimated crystallite sizes are in agreement with TEM imaging (Supplementary Fig. 3 and Supplementary Table 2). High-resolution TEM also confirms the cubic structure for the particles and indicates that no structural evolution has occurred during the functionalization reaction (Supplementary Fig. 4 and Supplementary Table 3). In addition, X-ray photoelectron spectroscopy (XPS) measurement at the Fe 2p edges shows an energy gap between 2p_1/2_ and 2p_3/2_ (13.7 eV), in agreement with the γ-Fe_2_O_3_ structure^35^ (Supplementary Fig. 5).

**Figure 2:**
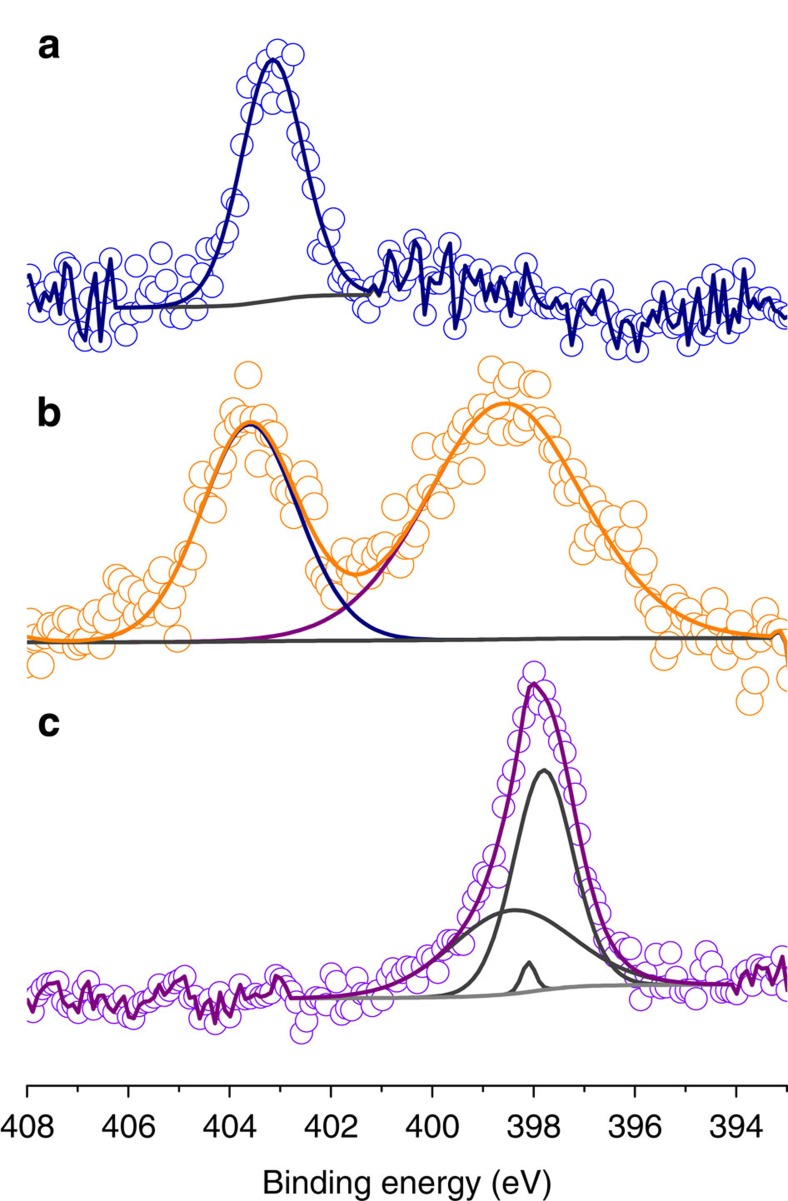
Presence of the enhancing unit in the functionalized maghemite nanoparticles. XPS spectra at the N1s edge of samples **0b** (**a**), **1** (**b**) and of the [Co(TPMA)Cl_2_] complex (**c**).

XPS measurements at the N 1s edge show two peaks at 404 and 399 eV for **1** (Fig. 2). The spectra of the bare nanoparticles **0b** and of the complex display only one peak at 403 and 398 eV, respectively. As the presence of nitrogen atoms can originate from the TMA^+^ counterions in **0b** and in **1**, and from the TPMA ligand in **1** and in [Co(TPMA)Cl_2_], the low energy contribution at 399 eV can be assigned to the nitrogen atoms from the TPMA ligand and the high energy peak at 404 eV to the contribution from the TMA^+^ counterion. The experimental Fe/N_lig_ atomic ratio of the peaks has been found equal to 1.00 for sample **1**, which differs from the calculated one (10 accounting for the TPMA ligands only). Nevertheless, the experimental Fe/N_lig_ ratio agrees well with the calculated one if only surface iron ions (∼10%) are taken into account. Atomic absorption spectroscopy (AAS) measurements made on a precipitated sample of **1** confirm the presence of cobalt(II) ions. The found 46±4 Fe/Co ratio corresponds to 52 complexes per particle (considering 2418 Fe(III) ions for a spherical 5 nm γ-Fe_2_O_3_ nanoparticle). This would indicate an 86% grafting rate corresponding to a surface density of 0.66 complex per nm^2^.

The presence of complexes coordinated to the nanoparticles surface has been further evidenced by X-ray absorption spectroscopy (XAS) measurements at the *L*_2,3_ edges of the iron and cobalt ions for sample **1** and at the cobalt *L*_2,3_ edges for the [Co(TPMA)Cl_2_] complex. For **1**, the spectra at the cobalt edges confirm the presence of octahedral Co(II) and the absence of Co(III) (Supplementary Fig. 6). Moreover, differences are observed between the spectra of **1** and of the ‘ungrafted' complex [Co(TPMA)Cl_2_]. They can be attributed to a change in the first coordination sphere of the cobalt ion, since we expect the replacement of chloride ions by oxo ligands through the condensation at the nanoparticle surface. Indeed chloride ligands are expected to induce a weaker ligand field than the oxo groups from the particle surface^36^. This is confirmed by ligand field multiplet calculations of Co *L*_2,3_ edges that indicate a ligand field in **1** stronger than in the [Co(TPMA)Cl_2_] complex (Supplementary Fig. 7 and Supplementary Methods).

In summary, the combination of XPS, AAS and XAS measurements clearly confirms the presence of the {Co^II^(TPMA)}^2+^ complex at the surface of the nanoparticles and the formation of an oxo-bridge between Co(II) and Fe(III) ions.

### Magnetic characterization

To assess the influence of the molecular complex and investigate the magnetic properties of the functionalized nanoparticles we have performed d.c. magnetization measurements, as well as Mössbauer and X-ray magnetic circular dichroism (XMCD) spectroscopies. Where the former gives the macroscopic behaviour of the functionalized nanoparticles, the latter two—as local probes—give element-specific information.

**Figure 3:**
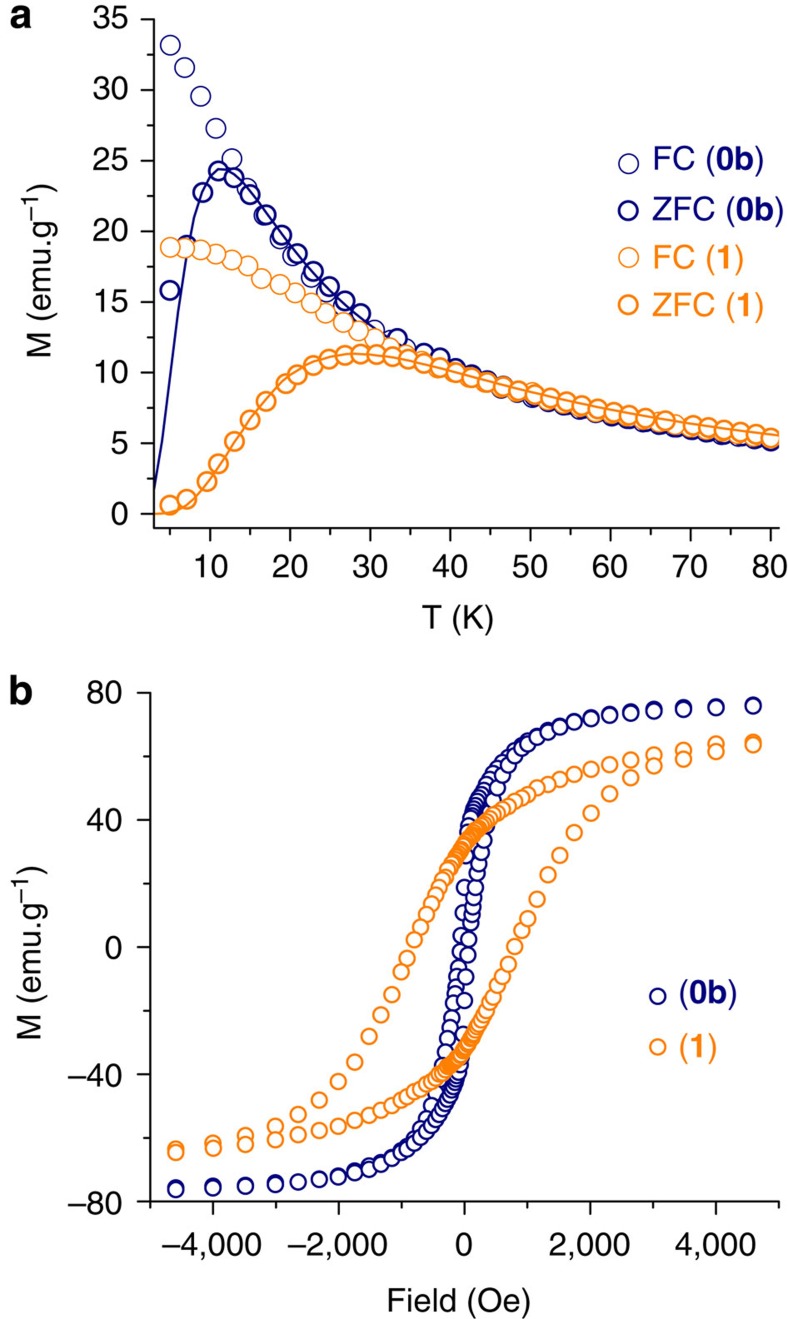
Enhanced anisotropy and improved magnetic properties. (**a**) Field-cooled and zero-field-cooled (FC/ZFC) magnetization curves measured in the 5–80 K temperature range under an applied field of 50 Oe and (**b**) magnetization vs field curves measured at 5 K for **0b** and **1** in diluted solutions (%v<0.15). Lines in the ZFC plots represent the best fit (see Methods for calculation details).

In **1**, the presence of the {Co^II^(TPMA)}^2+^ complexes at the nanoparticles surface increases considerably the temperature of the maximum in the zero-field-cooled (ZFC) magnetization curve, reaching 30 K (11 K for **0b**, Fig. 3). The fit of the ZFC curves gives—using the same size distribution function—effective anisotropy constants of 26 and 65 kJ m^−3^ for **0b** and **1**, respectively, attesting thus the anisotropy enhancement (Fig. 3a, see methods for calculation details). This enhancement is also confirmed with the magnetization vs field curves. No break in the hysteresis curve around the remnant magnetization is observed, in agreement with a uniform reversal of the magnetization (Fig. 3b and Supplementary Fig. 8). The presence of the complexes impressively increases the coercive field of the nanoparticles, multiplying the value by 13 (from 62 Oe for **0b** to 839 Oe for **1**). In an attempt to differentiate the effect of a surface modification due to the coordination of the complexes from that of a magnetic coupling between the Co(II) complexes and the nanoparticle, a Zn(II) analogue of **1** has been prepared and measured (**2**). The same quantity of the diamagnetic {Zn^II^(TPMA)}^2+^ fragment grafted on the particle surface does not induce a comparable effect on the temperature of the maximum in the zero-field-cooled magnetization curve (from 11 to 14 K; Supplementary Fig. 9). In the magnetization vs field curve, the presence of the {Zn^II^(TPMA)}^2+^ complex has a slight effect on the remnant magnetization but an almost negligible one on the coercive field (from 62 to 73 Oe; Supplementary Fig. 10). These results indicate that the effect of the {Co^II^(TPMA)}^2+^ units on the magnetic properties does not originate from a simple modification of the environment of the iron ions located at the nanoparticles surface. Moreover, since no aggregation of the nano-objects occurs after the condensation of the complexes, the observed effect necessarily results from the magnetic interaction of the complexes with the particles, leading to an increase of the magnetic anisotropy. The transmission of the anisotropy from the complexes to the particles is possible only if there is an exchange interaction between the Co(II) and the Fe(III) ions. As the observed enhancement of the magnetic properties is important and effective at relatively high temperature, electrostatic interactions must be ruled out. Only the occurrence of a chemical bond such as an oxo-bridge between the Co(II) and the Fe(III) ions can support the effective anisotropy enhancement, source of the improved properties.

**Figure 4:**
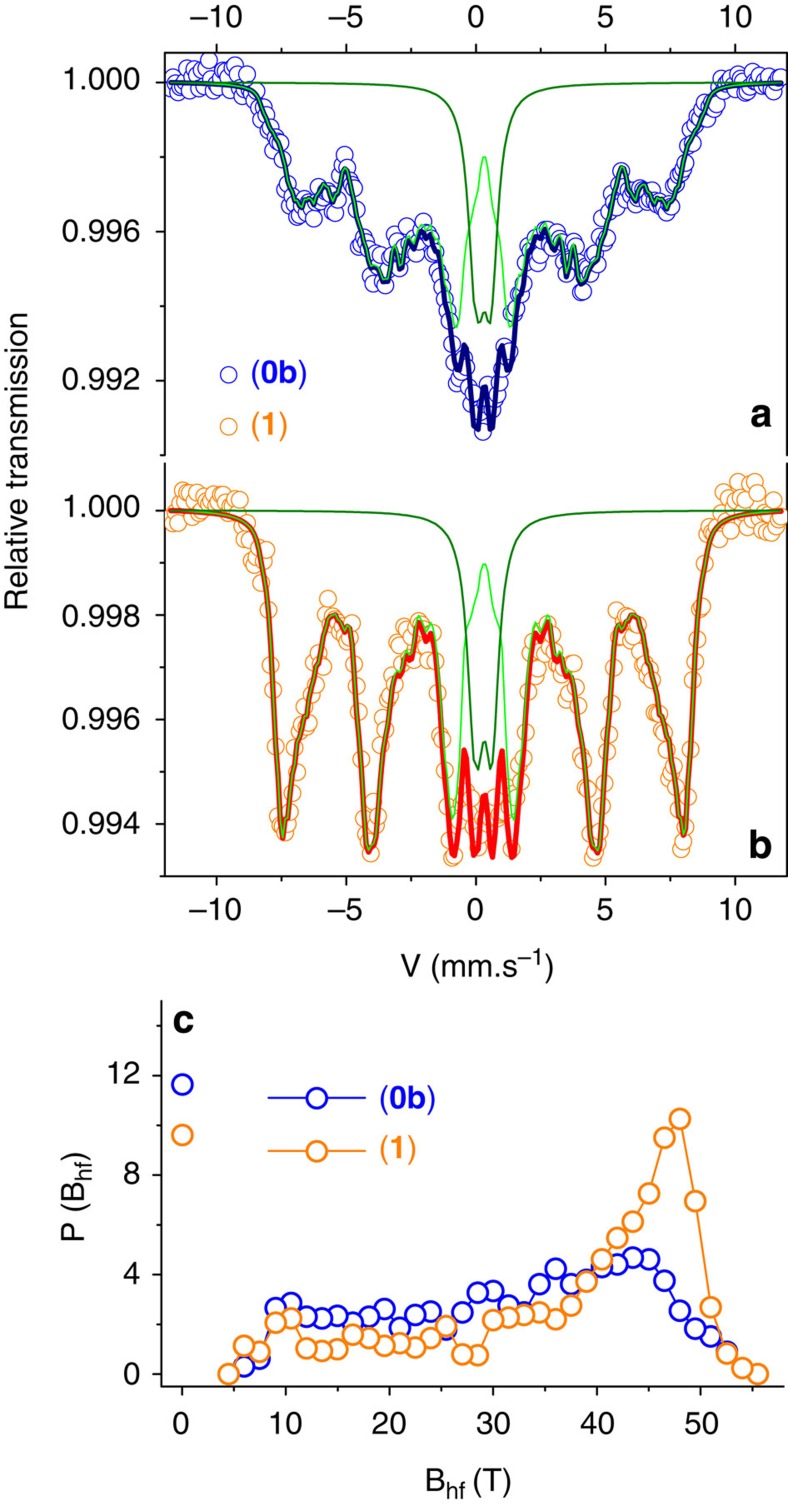
Slowdown of the relaxation of the magnetization. Zero field ^57^Fe Mössbauer spectra (circles: experimental; lines: calculated) measured at 77 K for **0b** (**a**) and **1** (**b**) and corresponding hyperfine field distributions (*P(B*_hf_)) vs hyperfine field (*B*_hf_) plot (**c**).

^57^Fe Mössbauer spectrometry has been performed at 77 K on frozen solutions of **0b** and **1** (Fig. 4 and Supplementary Fig. 11) to discriminate the chemical environment and magnetic properties of the different Fe species, through the analysis of the hyperfine interactions^37^. Indeed, this local probe technique remains a powerful tool for investigating Fe-containing nanoparticles and the influence of the functionalization, thanks to its high sensitivity to electron transfer^38^. The 77 K spectra result from a minor central quadrupolar doublet and a prevailing broadened lines magnetic sextet: they have exactly the same isomer shift and their proportions are rather independent of the samples. These two contributions are unambiguously assigned to Fe species with fast and weak superparamagnetic relaxation phenomena, due to size distributions in the samples. The lack of resolution does not allow the proportions of iron in tetrahedral and octahedral sites to be estimated but they were accurately estimated from in-8 T field Mössbauer spectra at 12 K (Fe^Oh^(III)/Fe^Td^(III)=1.70 close to 5/3 as expected for maghemite; Supplementary Fig. 11). The mean values of isomer shift (at 77 K 0.41(2) mm s^−1^), which probes the electronic density at the ^57^Fe nuclei, that is the valence state, are consistent with the presence of pure ferric species for both **0b** and **1.** This excludes the presence of a ferric impurity and the occurrence of Fe^2+^ species or intermediate valence state. It further evidences that no electron transfer is induced by the presence of the Co(II) complexes. The mean hyperfine field distribution profiles, which correspond to the shape of the magnetic lines, indicate clearly that the grafting of the complexes gives rise to both a shrinkage of the distribution and a shift towards larger hyperfine fields, that is a significant increase of the mean hyperfine field (28.4(5) and 35.1(5) T, respectively). These features distinctly attest a slowdown of the relaxation phenomena of the magnetization in **1** because the attached Co(II) complexes increase the magnetic anisotropy of the Fe(III) moments, strengthening thus the magnetization of each nanoparticle, in agreement with the ZFC measurements.

**Figure 5:**
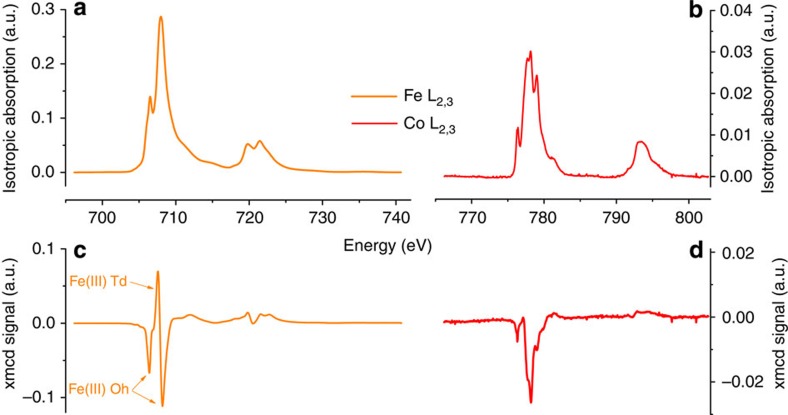
Element-specific characterization of the functionalized nanoparticles. XAS and XMCD signals measured on sample **1** at the Fe (**a**,**c**) and Co (**b**,**d**) *L*_2,3_ edges at 5 K and 6 T.

**Figure 6:**
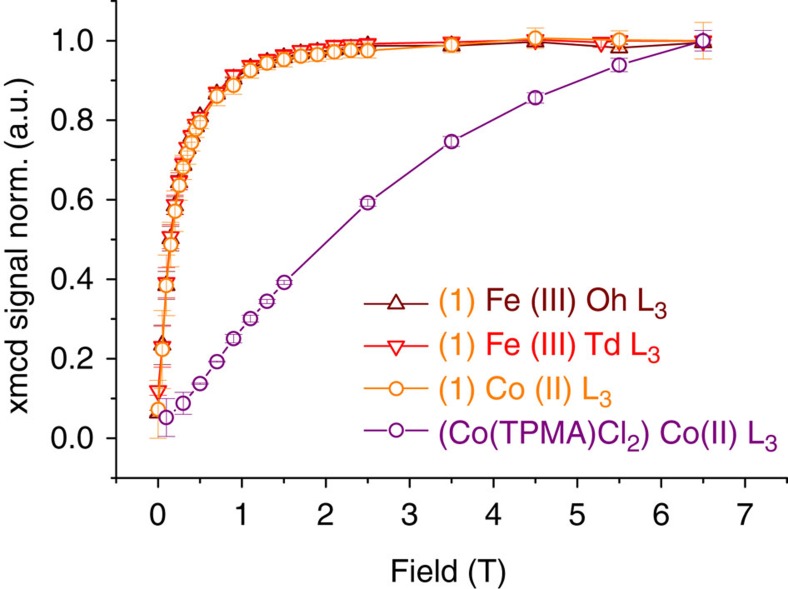
Fe-specific and Co-specific XMCD-detected magnetization curves at 5 K. The XMCD curves for octahedral Fe(III) and for Co(II) were multiplied by −1 before normalization. All the curves were normalized to one at the highest field value, error bars are s.d.

The shape and intensity of the XMCD signals at the Fe *L*_2,3_ edges for **1** are similar to those observed for previously reported maghemite nanoparticles^39^. It bears the signature of antiferromagnetic coupling between Fe(III) ions in tetrahedral sites and Fe(III) ions in octahedral sites (Fig. 5). The magnetic moment for Fe(III) ions in the sub-network of the octahedral Fe(III) is parallel to the external magnetic field. The ratio between the occupation of the tetrahedral and octahedral sites can be determined from the ligand field multiplet analysis of the XMCD shape and a Fe^Oh^(III)/Fe^Td^(III) ratio close to 5/3 is found, as expected for maghemite. Traces of Fe(II) have also been detected. The latter are due to sample preparation (see methods). The XMCD at Co *L*_2,3_ edges in **1** is mainly negative at the *L*_*3*_ edge indicating that the Co(II) magnetic moment is, at 6 T, parallel to the octahedral Fe(III) ions and antiparallel to the tetrahedral Fe(III) ions. Element-specific magnetization curves for Fe and Co were also obtained measuring the dependence of the XMCD signal as a function of the applied magnetic field amplitude (see methods). The Co-specific magnetization curve (Fig. 6 and Supplementary Fig. 12) does not show any inversion in the sign of the XMCD when varying the magnetic field, indicating that no inversion of coupling can be expected at low magnetic field. All three curves are superimposed demonstrating that the Co(II) is magnetically coupled to the Fe(III) ions of the maghemite nanoparticle. Moreover, the Co-specific magnetization curve of **1** differs drastically from the XMCD-detected magnetization curve of the [Co(TPMA)Cl_2_] complex. The latter shows a slow increase of the magnetization with no saturation reached at 6.5 T, as expected for a non-interacting paramagnetic Co(II) ion. For **1**, the magnetization increases abruptly and saturates above 2 T. This behaviour evidences and confirms that the Co(II) ions within the grafted complexes are magnetically coupled to the iron(III) ions at the nanoparticles surface.

## Discussion

We have presented in this work a synthetic strategy, which, in combining molecular and nano chemistry, offers a way towards control and modulation of the magnetic anisotropy in nanoparticles. Magnetic measurements, Mössbauer spectrometry and XMCD measurements show that {Co^II^(TPMA)}^2+^ complexes grafted on the surface of maghemite nanoparticles massively enhance the magnetic properties of the nano-objects. Our results also indicate that the strong influence of the molecular component on the nanoparticle comes from the covalent linking of the two species through oxo-bridges and the resulting magnetic interaction.

This work may open tremendous prospects in the design of nanomagnets and of multifunctional nano-platforms. Provided that the choice of nanoparticle to functionalize allows the formation of a coordination bridge able to promote magnetic exchange, and that the particle size and the characteristics of the molecule are adequately matched, it should be possible to obtain composite nano-objects with desired blocking temperature and coercive field. Objects prepared in soft conditions, in air, in aqueous media, and in the lack of surfactant to stabilize the colloidal solution represents an important advantage for the preparation of applied materials (surface deposition, biocompatible polymers). The possibility of performing chemistry on the ligand born by the complex also represents an asset and offers many additional possibilities. The adequate choice of ligand could easily allow pre- or post- functionalization, whether to add a property (organic chromophores) or to structure the objects (polymerizable/‘clickable' units) towards polyfunctional devices.

## Methods

### Preparation of **0a**

The solution was prepared according to literature procedures^33^,^34^ (5.1 nm, *σ*=0.12, [Fe]=0.87 M, %m=6.96%, %v=1.39%, pH=1.8).

### Preparation of **0b**

A measure of 250 μl of **0a** were diluted 10 times with a H_2_O:MeOH 50% v/v mixture. Then, 500 μl of an aqueous TMAOH solution (2.8 M) were brutally added to the solution under strong stirring leading to the sample **0b**.

### Preparation of **1**

A measure of 250 μl of **0a** were diluted 10 times with a H_2_O:MeOH 50% v/v mixture. A volume of 0.550 ml of a [Co(TPMA)Cl_2_] solution (10 mM, H_2_O:MeOH 50% v/v) were added dropwise under stirring, followed by the rapid addition of 500 μl of an aqueous TMAOH solution (2.8 M) under strong stirring. Then, the solution was stirred for 4 h at 60 °C and for 24 h at room temperature leading to sample **1**.

### Preparation of **2**

The sample was prepared following the procedure described for **1** using [Zn(TPMA)Cl_2_] instead of [Co(TPMA)Cl_2_].

### Precipitation of the particles

The addition of three volumes of acetone into the solutions led to the precipitation of the particles. The suspension was placed on a NdFeB magnet to settle the particles and the supernatant was removed. The obtained paste-like solid was washed with an aliquot of ethanol and dried in an oven at 40 °C for 48 h.

### Atomic absorption spectroscopy

The total iron, cobalt and zinc concentration (mol l^−1^) was determined by AAS with a Perkin–Elmer Analyst 100 apparatus after degrading the precipitated particles in HCl (37%).

### Transmission electron microscopy

Images have been performed on a JEOL 100CX2 microscope with 65 keV incident electrons focused on the specimen. High-resolution TEM has been achieved on a JEOL JEM 2011 microscope with an acceleration voltage of 200 kV and a resolution of 0.18 nm.

### Dynamic light scattering

The DLS measurements have been performed on a Malvern Zetasizer nanoZS model equipped with a backscattering mode on the solutions containing the particles using the intensity profile. The sizes given in the article correspond to the *Z* average measurements.

### X-ray powder diffraction

Patterns were collected on a Philips X'pert Pro diffractometer using Co-Kα1 monochromatic radiation (*λ*=1.78901 Å) and equipped with a X'celerator linear detector.

### Magnetic measurements

Magnetic measurements were carried out with Quantum Design MPMS-XL and MPMS-5S magnetometers working in d.c. mode on frozen solutions of the samples. The solution was diluted in a H_2_O:MeOH 50% v/v mixture before measurements. The solution volume was 150 μl and the weight concentration 0.4%. The solution is placed in a 0.2 ml eppendorf and inserted in the cryostat of the superconducting quantum interference device (SQUID) magnetometer and frozen directly from room temperature to 100 K in zero magnetic field (lowering the rod takes a few seconds) before any measurement. The temperature sweeping rate for ZFC/FC measurements was 2 K min^−1^.

### Anisotropy constants calculations

Following Tamion *et al*.^40^ we have used their semi-analytical model to describe the temperature dependence of the ZFC magnetization and extract an estimate of the magnetic anisotropy energy density *K*_eff_.

Having defined a switching-field frequency:


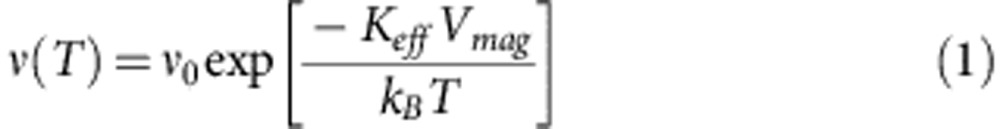


with *v*_0_=10^9^ Hz the attempt frequency and a characteristic time *δ*_*t*_*(T)*, which depends on the temperature sweeping rate (here 2 K min^−1^), the magnetic moment measured during a ZFC protocol is given by


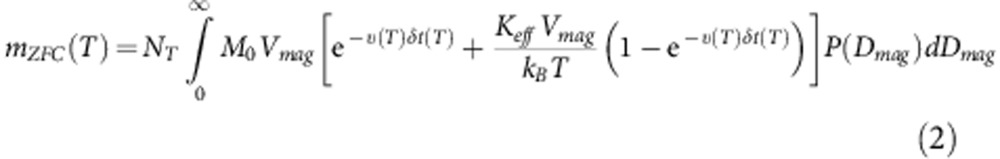


where *M*_0_*V*_mag_*=μ*_0_*m*_s_^2^*H/*(3*K*_eff_*V*_mag_) (equation 3) is the initial ZFC susceptibility in the frozen low temperature state and *P(D*_mag_) is the size distribution function taken here as a lognormal with characteristic parameter taken from the TEM characterization of the particles. In the equations above, *μ*_0_ and *k*_B_ denote the magnetic permeability of vacuum and the Boltzmann constant, *H* is the magnetic field strength, *m*_S_ is the saturation magnetization of the maghemite and *N*_*T*_ is the number of magnetically active clusters.

Using this equation, it is quite straightforward to obtain a calculated ZFC curve and optimize the value of *K*_eff_ in order that make it best match the experimental data.

### ^57^Fe Mössbauer spectrometry

^57^Fe Mössbauer spectra were performed at 77 K using a conventional constant acceleration transmission spectrometer with a ^57^Co source (Rh matrix) and a bath cryostat and at 12 K in a 8 T external field applied parallel to the γ-beam in a cryomagnetic device. The spectra were fitted by means of the MOSFIT program and an α-Fe foil was used as the calibration sample.

### X-ray photoemission spectroscopy analyses

At various times of adsorption, *t*=30 s, 1, 3 and 30 min, the chamber was evacuated to some 10^−10^ torr, and the sample was analysed by X-ray photoemission spectroscopy using an Omicron NanoTechnology GmbH (Taunusstein, Germany) Argus hemispherical analyser and a monochromatic AlKα X-ray source (1,486.6 eV). After recording a broad range spectrum (pass energy 100 eV), high-resolution spectra were recorded for the N 1*s*, C 1*s*, O 1*s* and Fe 2*p* core levels (pass energy 20 eV). High-resolution XPS conditions have been fixed: ‘sweep' analysis mode and an electron beam power of 280 W (14 kV and 20 mA). The spectra were fitted using the Casa XPS v.2.3.16 Software (Casa Software Ltd., UK) and applying a Gaussian/Lorentzian ratio G/L equal to 70/30.

### XAS and XMCD measurements

XAS and XMCD spectra at Fe and Co *L*_2,3_ edges were recorded on the soft X-ray beamline DEIMOS^41^ at synchrotron SOLEIL (France). Circularly polarized photons delivered by an Apple II undulator are monochromatised by a variable groove depth (VGD) grating monochromator working in the inverse Petersen geometry. All reported spectra were measured using total electron yield detection under a 10^−10^ mbar ultra-high vacuum (UHV). The XMCD signals were recorded by both flipping the circular polarization (either left or right helicity) and the applied magnetic field (either +6 or −6 T). The XMCD signal is obtained as the difference *σ*_XMCD_=*σ*^−^−*σ*^+^ where *σ*^−^=[*σ*_L_(H^−^)+*σ*_R_(H^+^)]/2, *σ*^+^=[*σ*_L_(H^+^)+*σ*_R_(H^−^)]/2, *σ*_L_ (*σ*_R_) is the cross-section with left (right) polarized X-rays, and H^+^ (H^−^) the magnetic field parallel (antiparallel) to the X-ray propagation vector. This procedure ensured a high signal-to-noise ratio and allowed us to discard any spurious systematic signals. XAS and XMCD spectra were measured for samples cooled to 5 K and in a 6 T applied magnetic field.

The XMCD-detected magnetization curves are the field dependence of the dichroic signal. The XMCD amplitude is recorded at the energy of its maximum amplitude (707.56 eV for Td Fe, 708.19 eV for Oh Fe and 778.19 eV for Co) by quickly switching the circular polarization thanks to the electromagnet/permanent magnet helical undulator (EMPHU)^41^ available on DEIMOS beamline. Due to the presence of TMA^+^ cations, drop-casts of the nanoparticles solution on gold-coated silicon plate yielded highly hydroscopic deposits. The solid samples were thus prepared by precipitation in acetone. The solid was suspended in ethanol, drop-casted on a slide, dried on a hot plate and fixed on carbon conductive tape to the copper sample holder. Traces of Fe(II) have been detected on the XAS spectrum of **1** and estimated at ∼2% of the iron signal. This cannot be the result of an electron transfer from the Co(II) to the Fe(III) (Supplementary methods).

## Additional information

**How to cite this article:** Prado, Y. *et al*. Enhancing the magnetic anisotropy of maghemite nanoparticles *via* the surface coordination of molecular complexes. *Nat. Commun*. 6:10139 doi: 10.1038/ncomms10139 (2015).

## Supplementary Material

Supplementary Information Supplementary Figures 1-12, Supplementary Tables 1-3, Supplementary Methods and Supplementary References
