# Supplementary material for: Enhancing the magnetic anisotropy of maghemite nanoparticles via the surface coordination of molecular complexes
Source: Nat Commun. 2015 Dec 4;6:10139. doi: 10.1038/ncomms10139 (PMC4686836; doi:10.1038/ncomms10139)
Supplement: Supplementary Information — Supplementary Figures 1-12, Supplementary Tables 1-3, Supplementary Methods and Supplementary References [file ncomms10139-s1.pdf]

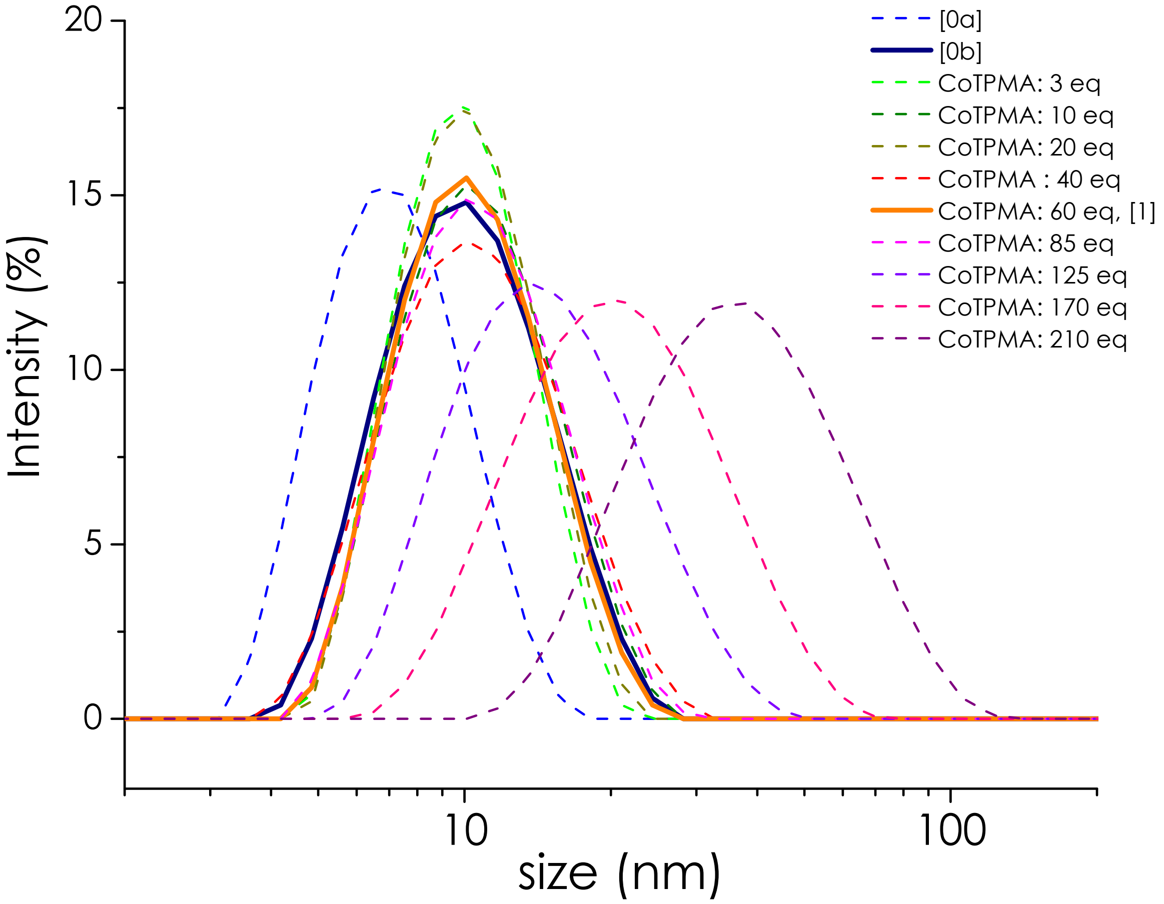

Supplementary Figure 1. Hydrodynamic size distribution in a H_2O:MeOH 50% v/v mixture at 25°C for the bare nanoparticles (**0a** and **0b**) and the nanoparticles functionalised with the complex $\{Co^{II}(TPMA)\}^{2+}$ in basic pH from Neq = 3 to Neq = 210.

a

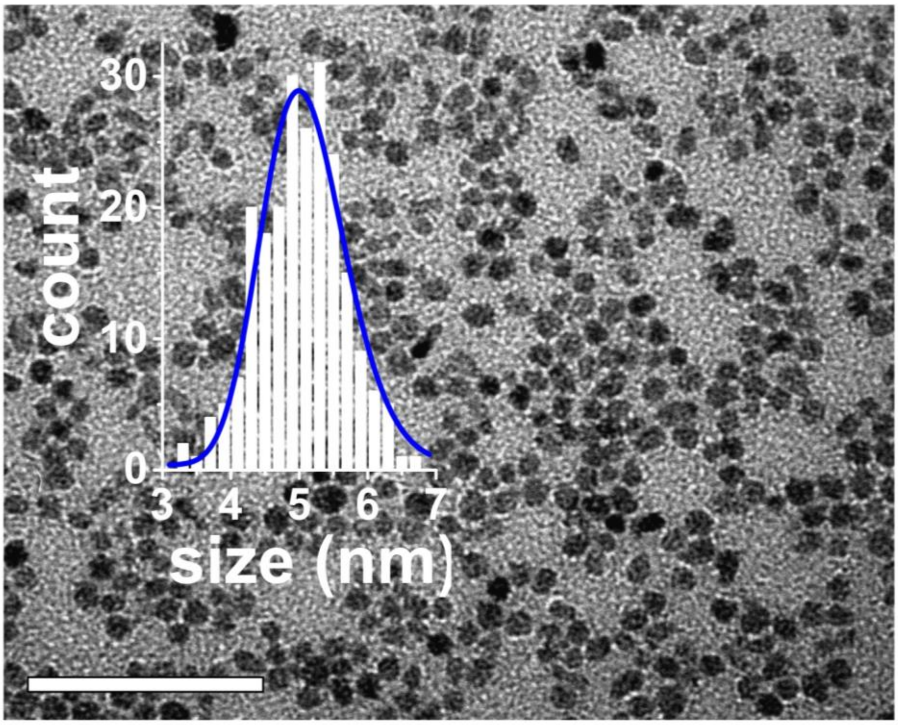

b

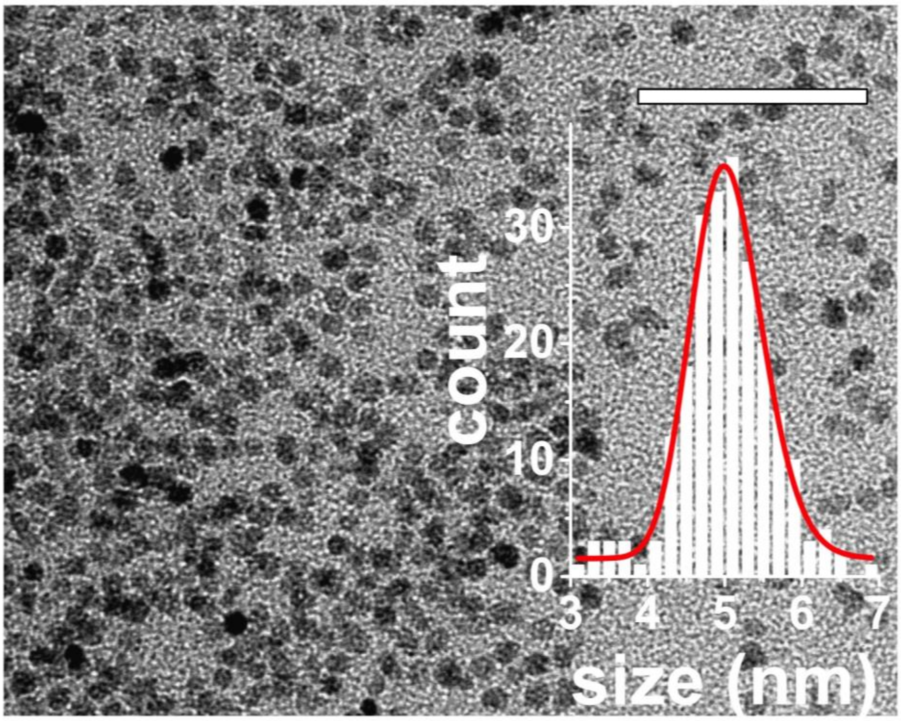

Supplementary Figure 2. TEM images of the bare nanoparticles **0b** (a) and of **1** (b), scales bar: 50 nm (insert: distribution made on approx. 200 nanoparticles and log normal fit).

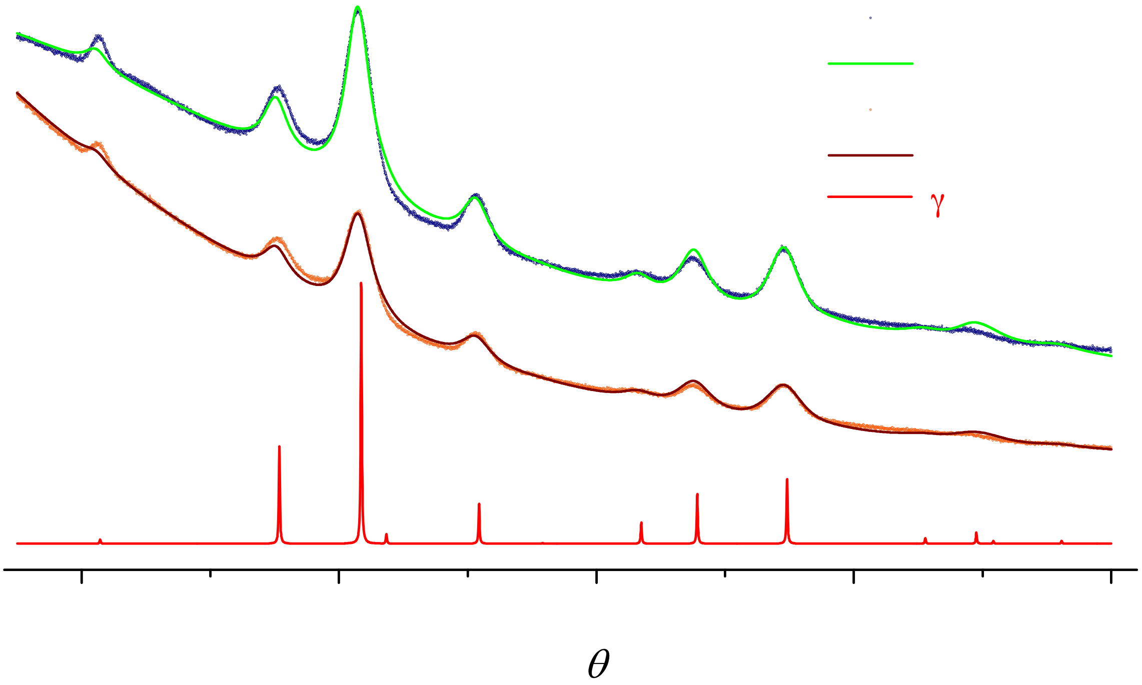

Supplementary Figure 3. X-ray powder diffraction patterns of **0b** and **1**, and calculated diagram for γ-Fe_2O_3 in *Fd-3m*.

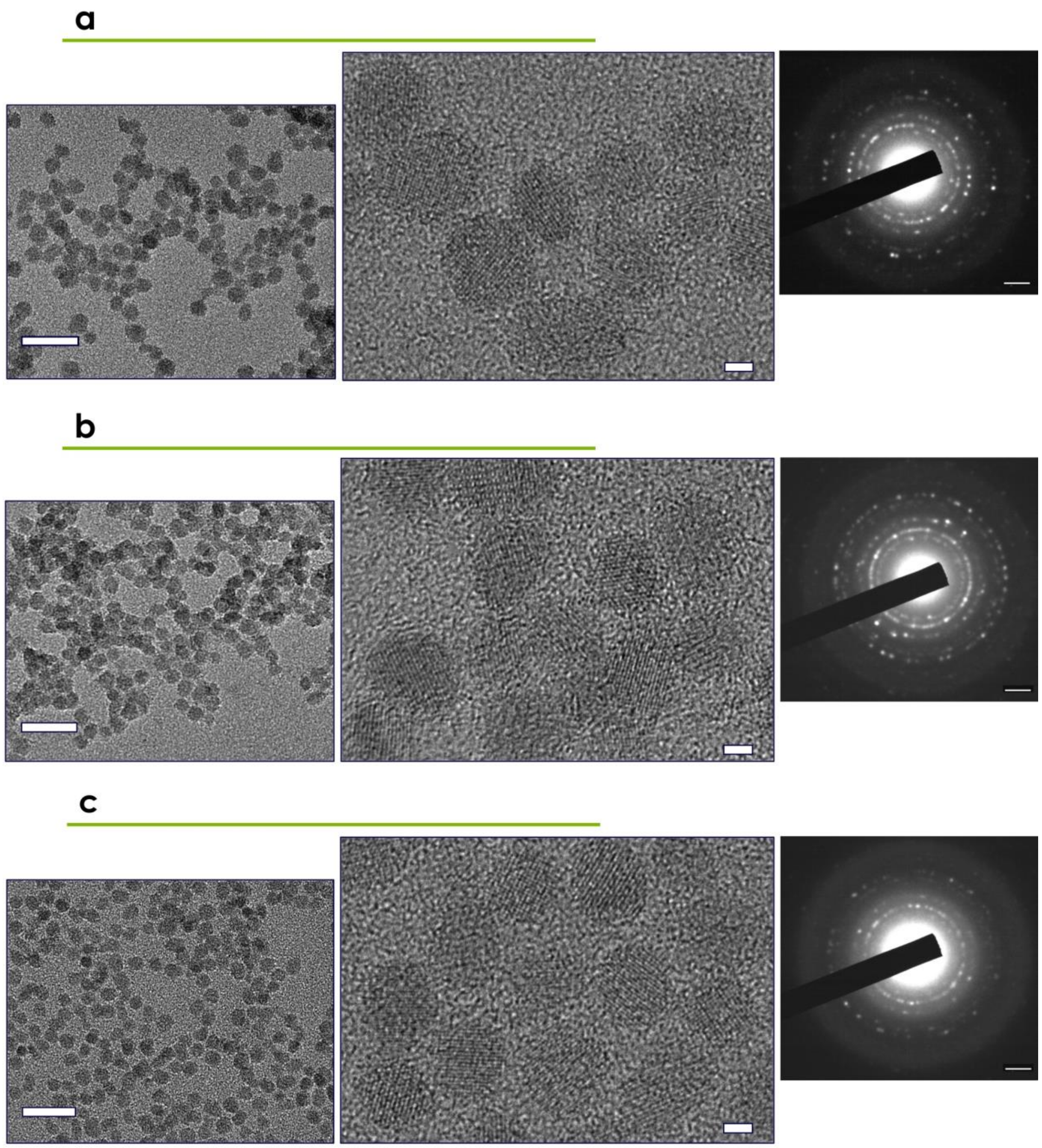

Supplementary Figure 4. TEM (scale bar 20 nm), HR-TEM (scale bar 2 nm) and SAED (scale bar 2 nm) images of the bare nanoparticles **0a** (a), **0b** (b) and of **1** (c).

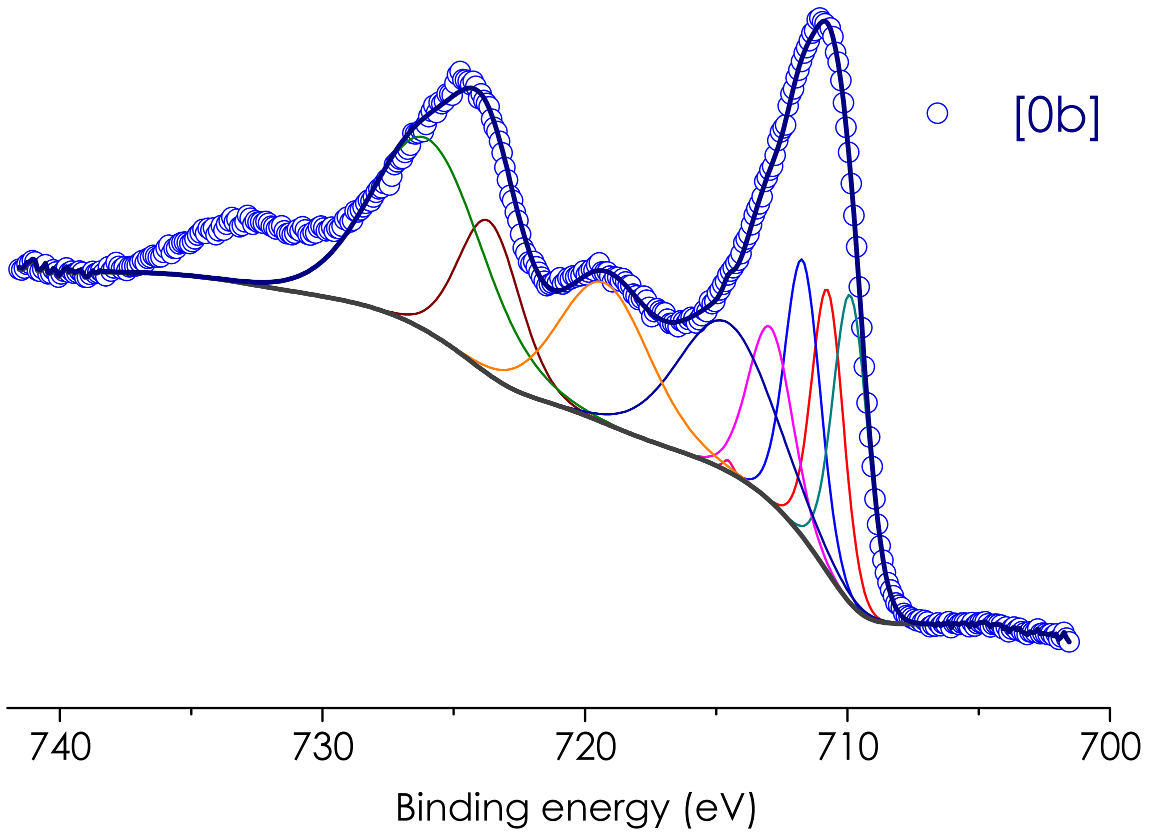

Supplementary Figure 5. XPS spectra at the Fe2p edge for sample **0b**.

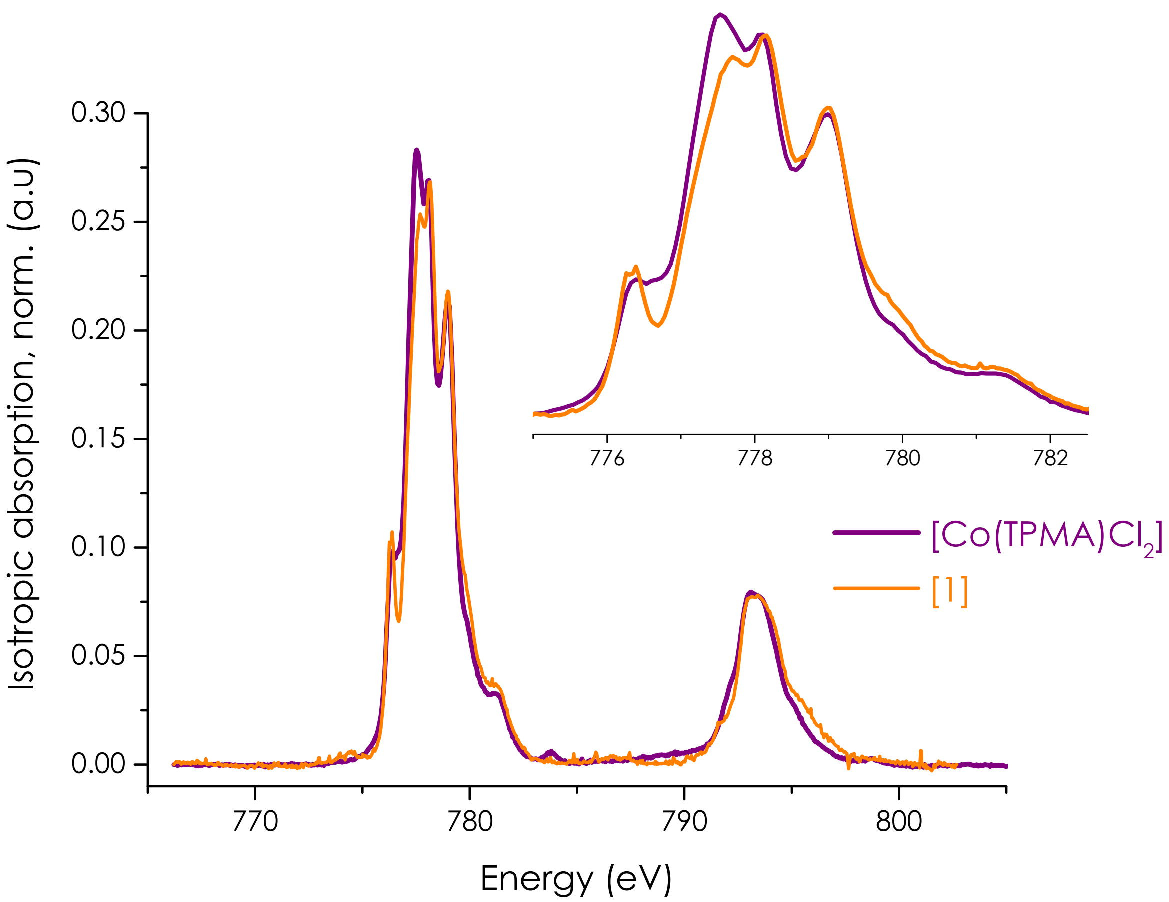

Supplementary Figure 6. XAS at Co $L_{2,3}$ edges for **1** and the [Co^{II}(TPMA)Cl$_2$] complex, measured at 5 K and 6 T.

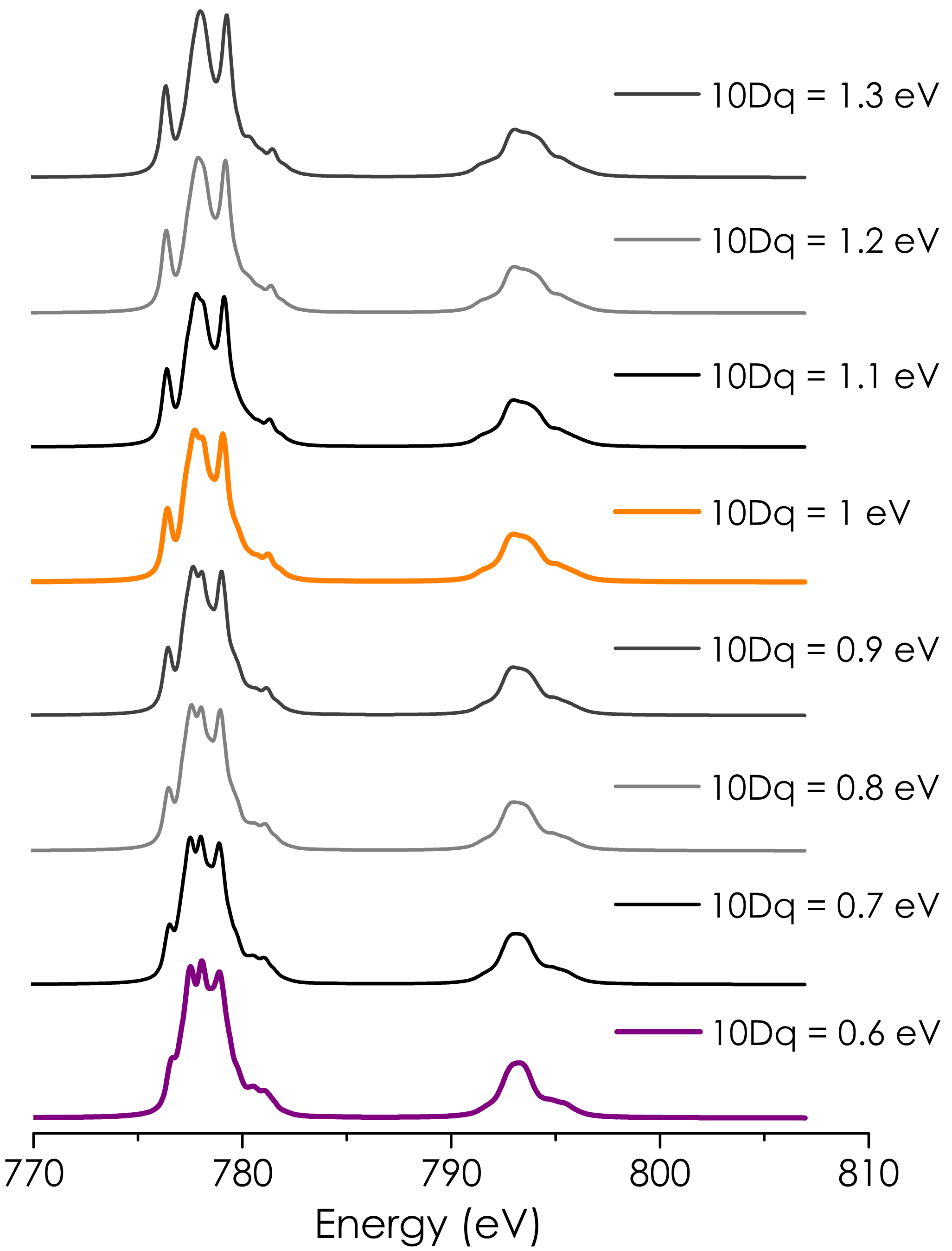

Supplementary Figure 7. Influence of the ligand field strength on the XAS spectra at Co $L_{2,3}$ edges calculated in the Ligand Field Multiplet model.

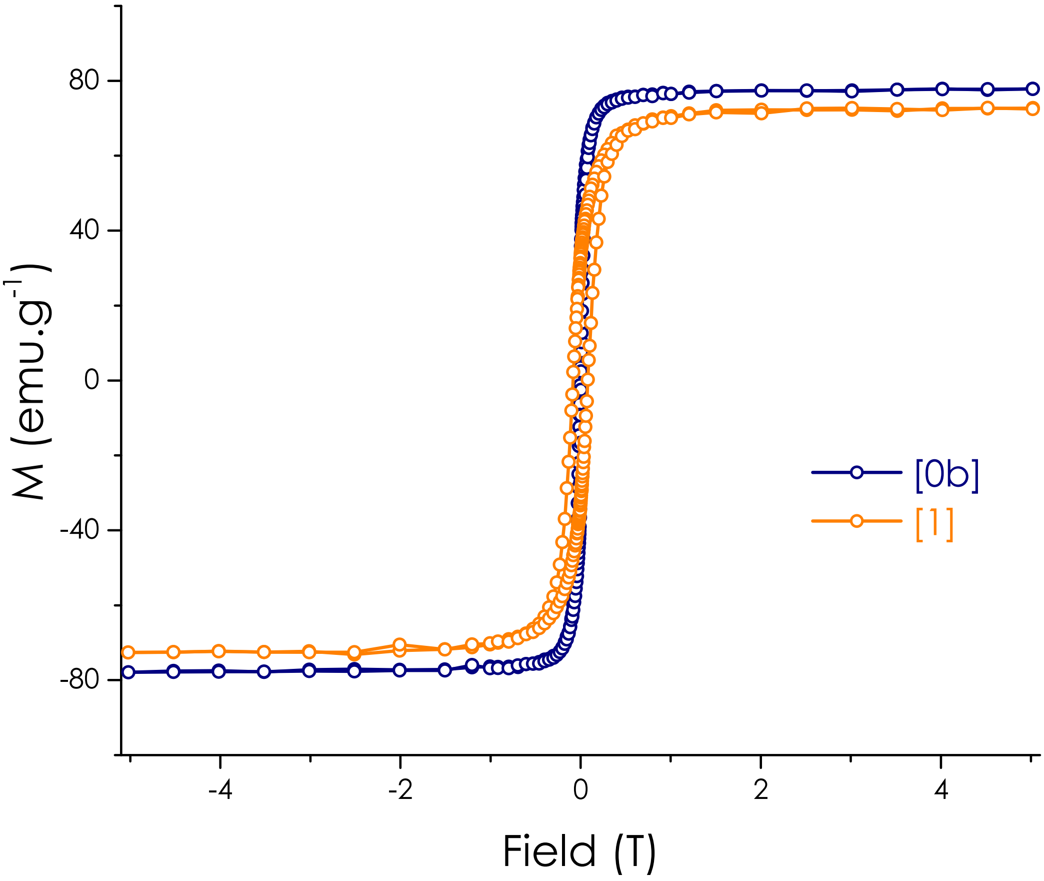

Supplementary Figure 8. Magnetisation vs. field curves for frozen solutions of **0b** and **1** at 5 K.

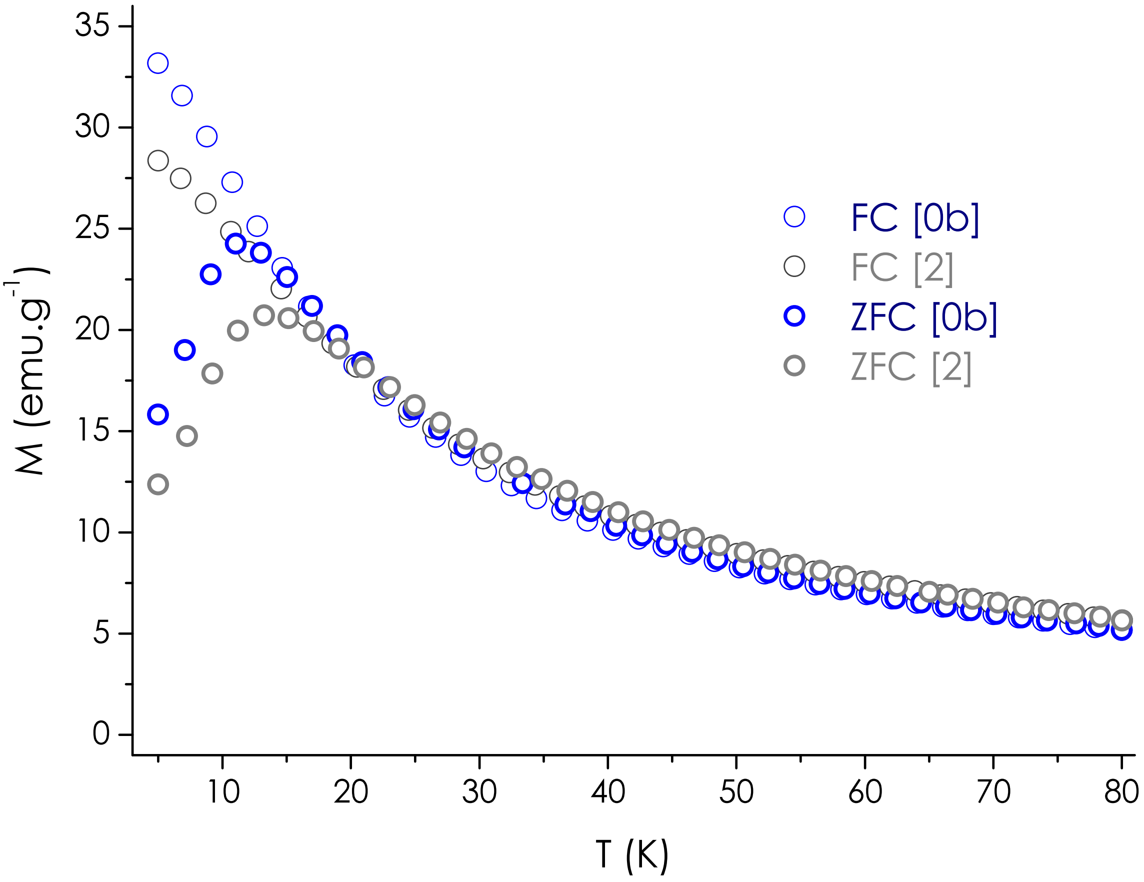

Supplementary Figure 9. ZFC/FC curves for a frozen solution of **2** under an applied field of 50 Oe compared to **0b**.

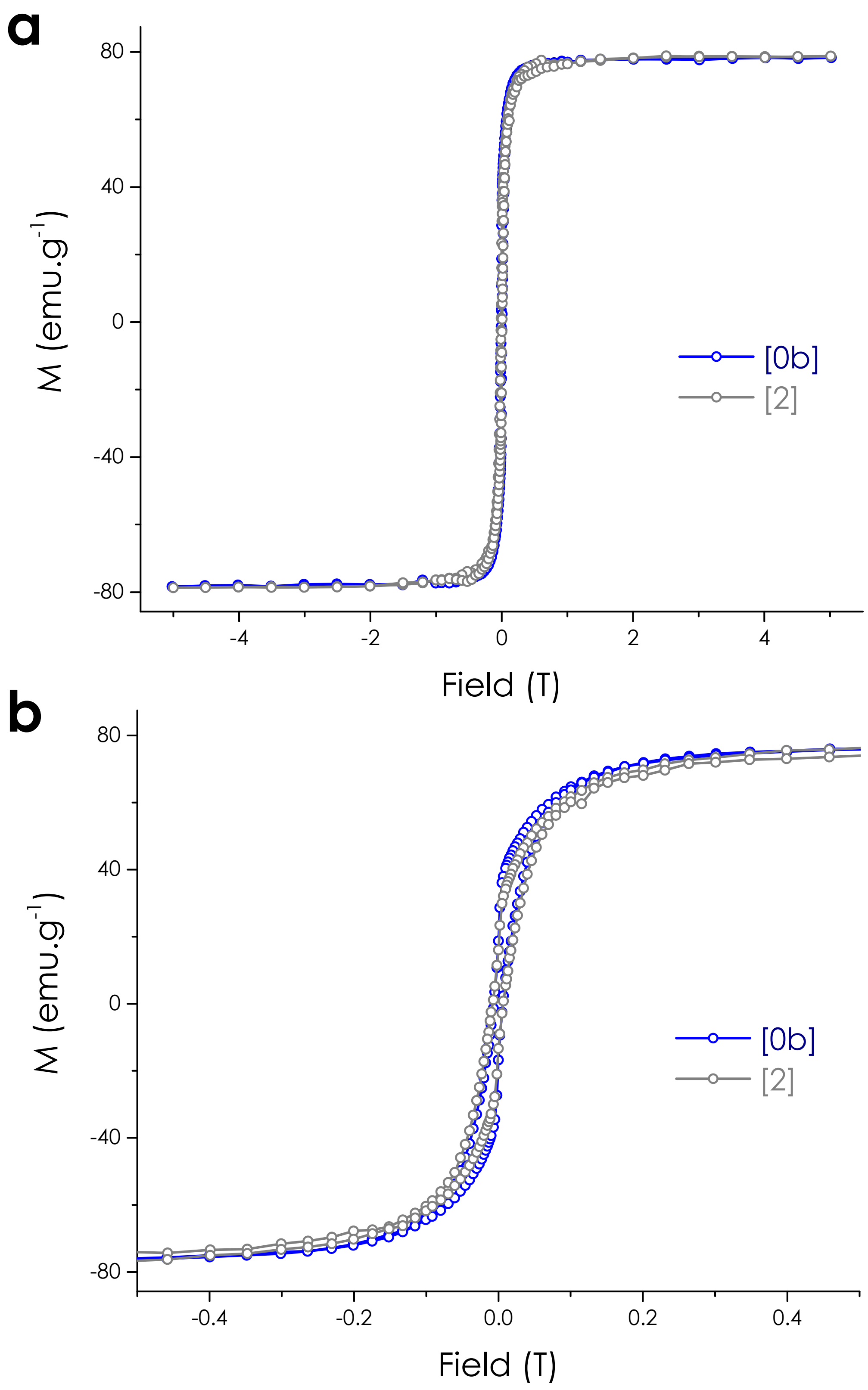

Supplementary Figure 10. Magnetisation vs. field curves for a frozen solution of **2** at 5 K compared to **0b**, full plot (a) and zoomed in between ± 0.5 T (b).

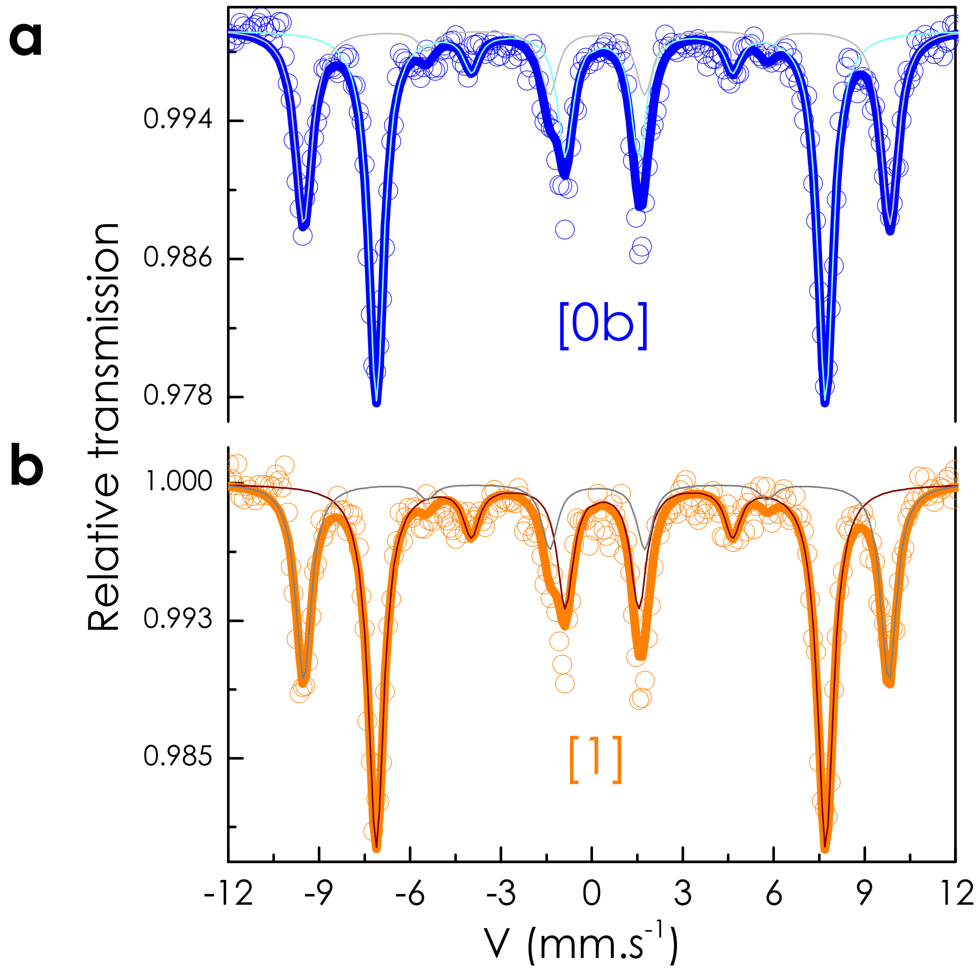

Supplementary Figure 11. ^{57}Fe Mössbauer spectra (circles: experimental; lines: calculated) measured at 12 K for **0b** (top) and **1** (bottom) in a 8 T external field. One can distinguish two different sextets: the external and internal ones are attributed to the tetrahedral and octahedral Fe sites, respectively.

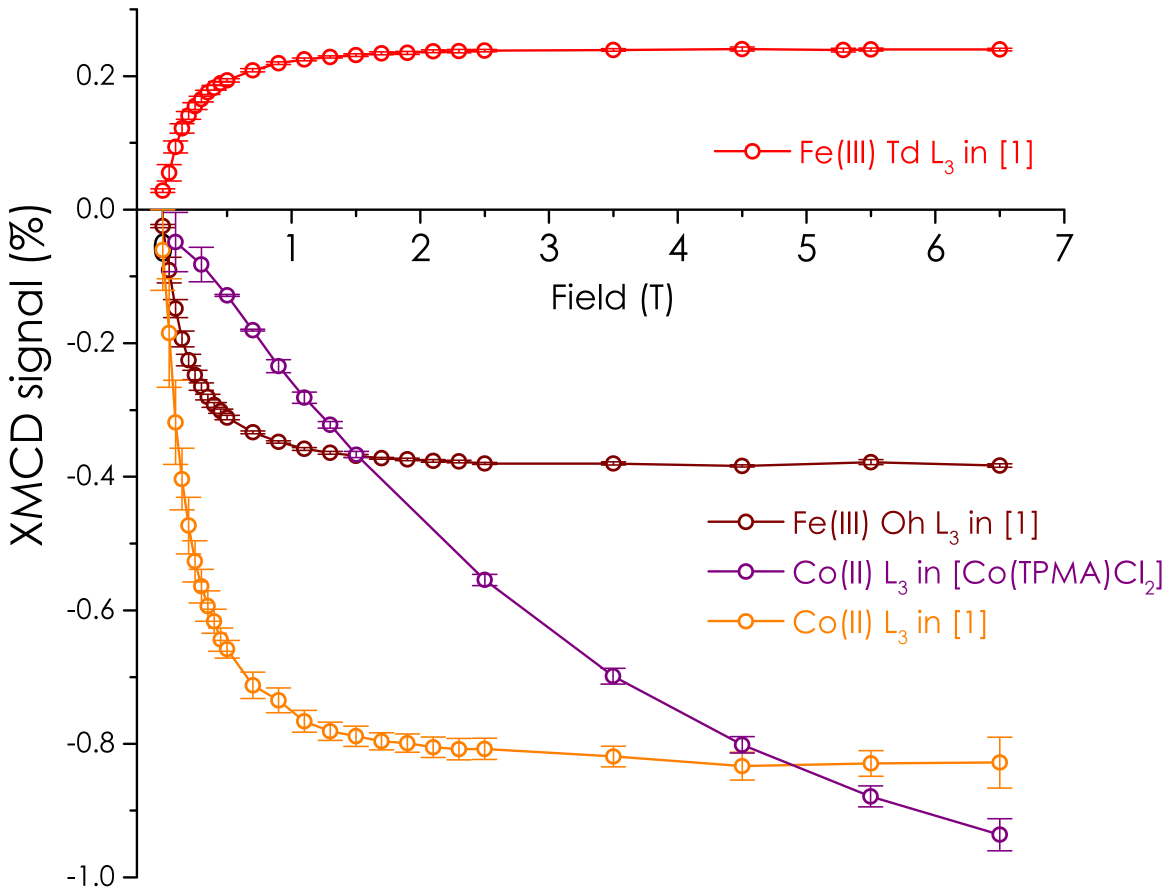

Supplementary Figure 12. Field-dependence of the XMCD signals at 5 K measured at 707.56 eV for tetrahedral Fe(III), 708.19 eV for octahedral Fe(III) and 778.19 eV for Co(II) in **1** and in [Co(TPMA)Cl_2]; error bars are s.d. The XMCD signal is given as the percentage of the maximum of the corresponding L_3 edge in the isotropic XAS spectrum. Necessarily, the total contribution for iron(III) is the sum of the iron(III) Oh and the iron(III) Td signals.

Supplementary Table 1. Hydrodynamic diameters extracted from the overall average size values (Z_{av}) in a H_2O:MeOH 50% v/v mixture at 25°C for the bare nanoparticles (**0a** and **0b**) and the nanoparticles functionalised with the complex $\{Co^{II}(TPMA)\}^{2+}$ in basic pH from Neq = 3 to Neq = 210.

	**[0a]**	**[0b]**	3	10	20	40	**[1]**	85	125	170	210
Z_{av} (nm)	7.3	9.5	9.9	10.2	9.9	9.7	10.2	10.2	14.1	18.3	32.2

Supplementary Table 2. Refinement results and crystallite size evaluation.

	Cell parameters a (**Å**)	Crystallite Size (**nm**)	R.m.s. microstrain
**0b**	8.349	5.6 (0.5)	0.003
**1**	8.346	5.6 (0.5)	0.004

Supplementary Table 3. Calculated d values for **0a**, **0b** and **1** (on 100 particles each) and theoretical values for maghemite[1].

	(hkl)	(420)	(430)	(620)	(800)	(753)
		d (Å^{-1})	d (Å^{-1})	d (Å^{-1})	d (Å^{-1})	d (Å^{-1})
Theoretical values for maghemite		0.1865	0.167	0.1318	0.1043	0.0963
Samples	[0a]	0.1935	0.1661	0.1369	0.1059	0.0968
	[0b]	0.1943	0.1649	0.1365	0.1056	0.0965
	[1]	0.1925	0.1643	0.1381	0.1054	0.0969

Supplementary Methods

X-ray powder diffraction. XRD patterns were fitted using Maud program (http://maud.radiographema.com/), which is based on the Rietveld method combined with Fourier analysis to describe the broadening of Bragg peaks.[2] This analysis allows the mean lattice parameters, the mean crystalline grain sizes to be estimated. The lack of agreement between experimental and theoretical curves may originate from the very small contribution of the absorption due to the sample compared to that of the substrate.

Ligand Field Multiplet Calculations. The theoretical $L_{2,3}$ edges spectra were calculated by using the Ligand Field Multiplet code developed by Thole[3], in the atomic and symmetry adapted framework established by Cowan and Butler[4,5]. This approach takes into account the multielectronic Coulomb repulsions, the 3d and 2p spin-orbit coupling, spin and orbit contributions to the Zeeman Hamiltonian and treats the geometric environment of the absorbing atom by an electrostatic potential[6]. The spectrum is calculated as the sum of all possible transitions between an atomic ground state $2p^6 3d^7$ configuration and all excited $2p^5 3d^8$ configurations with a core-hole on the 2p level. The interaction Hamiltonian between X-ray and matter is written in the electric dipole approximation. The electric dipole allowed 2p to 4s transitions are found negligible. Multielectronic Coulomb repulsions are modeled through the Slater Integrals calculated within an atomic Hartree-Fock model and the Slater integrals are scaled down by a κ reduction factor to account for the electronic delocalisation. The 3d spin-orbit coefficients are taken to 30 % of the Hartree-Fock calculated ones. In a first approximation, an octahedral O_h electrostatic ligand field describes the coordination sphere of the Co(II) absorbing ion. The ligand field strength is given by 10Dq. The calculated discrete line spectra are convolved with Gaussian function (HWHM = 1.18 σ eV) for instrumental broadening and a Lorentzian function (HWHM = 2Γ) with different Γ · · · · · s for L_3 and L_2 edges to account for super Coster-Kronig transitions. Figure S6 presents the XAS spectra calculated with κ=0.7, σ=0.1eV, Γ=0.2eV (=0.4eV) at L_3 edge (L_2 edge) and 10Dq varying from 0.6 eV to 1.2 eV. The experimental XAS of the complex [Co^{II}(TPMA)Cl_2] corresponds to the

calculation with a small ligand field strength, 10Dq=0.6eV, and the experimental XAS of **1** corresponds to the calculation with stronger ligand field, 10Dq=1eV.

Presence of Fe(II). Traces of Fe(II) have been detected on the XAS spectrum of **1** and estimated at ~ 2 % of the iron signal. This cannot be the result of an electron transfer from the Co(II) to the Fe(III). Indeed, the quantity of grafted cobalt complexes amounts for 2.2 % of the total iron ions. A reduction of the iron ions coordinated to the cobalt complexes at the nanoparticle surface would result in no cobalt(II) being observed. This is clearly not the case, and furthermore the XAS spectrum at the Co $L_{2,3}$ edge shows no evidence of Co(III).

We also believe that the traces of Fe(II) cannot result from a chemical reduction occurring during the synthesis of the nanoparticles. Indeed the functionalisation reaction takes place at pH 12 where the reduction reaction is unlikely. In addition no Fe(II) is evidenced from Mössbauer spectrometry. This could leave the precipitation of the nanoparticles and the preparation of solid samples as a potential source of undesired reduction recations. However, XPS shows pure maghemite. Thus we conclude that the particular solid sample preparation we have used for our XAS measurements, which differs from XPS, may have cause the occurrence of Fe(II).

Supplementray References

1. Cornell, R. M., Schwertmann, U. & Editors. *The Iron Oxides: Structure, Properties, Reactions, Occurrence and Uses.* (VCH, 1996).
2. Lutterotti, L. & Scardi, P. Simultaneous structure and size–strain refinement by the Rietveld method. *J. Appl. Cryst.* **23,** 246–252 (1990).
3. Thole, B. *et al.* 3d x-ray-absorption lines and the 3d94fn+1 multiplets of the lanthanides. *Phys. Rev. B* **32,** 5107–5118 (1985).
4. Cowan, R. D. *The Theory of Atomic Structure and Spectra.* (Univ. Calif. Press, 1981).
5. Butler, P. H. *Point Group Symmetry Applications: Methods and Tables.* (Plenum Press, 1981).
6. De Groot, F. M. F., Fuggle, J. C., Thole, B. T. & Sawatzky, G. A. The 2p x-ray absorption of 3d transition-metal compounds: an atomic multiplet description including the crystal field. *Phys. Rev. B: Condens. Matter* **42,** 5459–68 (1990).
